# Congenital Duodenal Diaphragm in a Toddler: A Case Report

**DOI:** 10.3390/reports8040251

**Published:** 2025-11-28

**Authors:** Maria Rogalidou, Chrysa Georgokosta, Palagia M. Karas, Konstantina Dimakou, Alexandra Papadopoulou

**Affiliations:** 1 Division of Gastroenterology and Hepatology, First Department of Paediatrics, National and Kapodistrian University of Athens, ‘Agia Sophia’ Children’s Hospital, 115 27 Athens, Greece; 2 First Department of Paediatrics, ‘Agia Sophia’ Children’s Hospital, 115 27 Athens, Greece; 3 Radiology Department, Agia Sofia Children’s Hospital, 115 27 Athens, Greece; 4 Gastroenterology Department, “Agia Sofia” Children’s Hospital, 115 27 Athens, Greece

**Keywords:** congenital duodenal diaphragm/web, duodenal membranous atresia, toddler, intermittent vomiting, failure to thrive (FTT)

## Abstract

**Background and Clinical Significanc:** Congenital duodenal diaphragm (CDD) is a rare congenital condition causing partial or complete obstruction of the duodenum, most frequently located in the second part of the duodenum. It is a rare but important cause of intestinal obstruction in infants and young children. Clinically, it often presents with persistent vomiting and failure to thrive. Diagnosis can be made through abdominal X-ray showing the characteristic “double bubble” sign, upper gastrointestinal (GI) series, or gastroscopy. **Case Presentation:** A 17-month-old female infant with known psychomotor retardation was admitted for evaluation of inadequate weight gain and intermittent postprandial vomiting, both present since birth. Laboratory investigations, including metabolic and electrolyte panels, were within normal limits. Given the persistent clinical symptoms, an upper gastrointestinal series was performed to assess for possible anatomical abnormalities. Imaging revealed a significant delay in the passage of contrast into the second portion of the duodenum, with marked prestenotic dilatation. Subsequent gastroscopy identified a duodenal diaphragm nearly occluding the duodenal lumen at the same site, impeding the passage of the endoscope. Associated findings included gastritis and the presence of food debris in the stomach and proximal duodenum, indicating impaired gastric emptying. The patient underwent successful surgical management via duodenotomy with resection of the septum. Postoperative recovery was uneventful. **Conclusions:** In infants or young children with persistent postprandial vomiting and inadequate weight gain, anatomical causes such as duodenal diaphragm/web should be considered in the differential diagnosis. Once identified, treatment should be initiated promptly, either endoscopically or surgically, depending on the severity and anatomical characteristics of the obstruction.

## 1. Introduction and Clinical Significance

A congenital duodenal diaphragm (CDD)/web, also referred to as duodenal mem-branous atresia, is a mucosal and submucosal membrane that partially or completely obstructs the duodenal lumen. It often features a central fenestration (“web”) or may elongate into a “windsock” shape due to peristalsis [1]. CDD arises from a failure of duodenal recanalization during embryogenesis, typically between the eighth and tenth weeks of gestation [1]. This is a rare pediatric surgical condition, with an estimated incidence of approximately 1 in 9000 to 40,000 live births [2]. Over 50% of cases are associated with other congenital anomalies, including Down syndrome, annular pancreas, congenital heart defects, malrotation, and prematurity [1]. Similarly to duodenal atresia, prenatal polyhydramnios occurs in approximately 33–50% of cases [1].

Clinical presentations include intermittent vomiting, failure to thrive (FTT), food intolerance, epigastric distension, or even vague abdominal pain. Older children may exhibit gastroesophageal reflux, ulcers, or strictures. Rarely, obstruction may occur from ingested foreign bodies or stones lodging in the web aperture, as seen with fruit seeds or pigmented stones. The degree of vomiting and the time of onset vary with the size and patency of the openings. Infants with smaller openings typically experience severe vomiting during their initial feeding after birth, necessitating early neonatal surgical intervention. Conversely, those with larger openings may present intermittent vomiting symptoms and delay seeking medical attention for months or even years following birth. The prognosis for a CDD post-surgery is generally favorable in the absence of concurrent severe malformations.

The diagnosis can be based on a plain abdominal X-ray and may show the classic double bubble sign, especially in neonates and young infants, but can be less obvious in partial obstructions [1]. Upper GI contrast studies (barium meal) are more sensitive, revealing proximal duodenal dilation, narrowing at the web, and the “windsock” silhouette [3]. Endoscopy can directly visualize the web, its location (often near the ampulla of Vater), and the characteristic bulging membrane. It is widely used in toddlers and older children [3].

Treatment approaches include the following:

- Surgical Management: Standard procedure is Duodenotomy with surgical excision of the diaphragm/web and closure (often transverse closure to prevent stricture) [4]. Intraoperative exploration for a second web is recommended [1].
- Endoscopic Management: Balloon dilation of the web has been successful in some children—e.g., an 8-year-old girl underwent serial dilations using CRE balloons (10 mm then 12 mm), which resolved vomiting and restored weight gain [3].
- In adult or older pediatric cases, endoscopic submucosal dissection (ESD)-like techniques have been used—e.g., resecting the diaphragm using fine instruments while avoiding injuring the ampulla of Vater [2].
- Endoscopic Electrocauterization (IT-knife): A 35-month-old child (with Down syndrome) had the web excised using an insulated-tip knife, avoiding more invasive surgery and recovering well [5].

The prognosis for a CDD post-surgery is generally favorable in the absence of concurrent severe malformations. With timely diagnosis and appropriate treatment, the prognosis is excellent, with full symptom resolution and normal growth following surgery or endoscopic intervention; even for delayed presentations, positive outcomes have been observed [6]. Early diagnosis and appropriate treatment, whether through traditional surgery or newer endoscopic techniques, are crucial for managing CDD in children [7,8].

We present a toddler with CDD, a rare cause of vomiting in childhood that, with early diagnosis by endoscopy and successful treatment with surgical intervention, has resulted in the resolution of symptoms and weight gain.

## 2. Case Presentation

A 17-month-old female was admitted to our hospital because of poor weight gain and recurrent episodes of projectile vomiting since birth. Pregnancy and birth history were unremarkable; she is the first child of healthy parents, Syrian refugees. She has a history of psychomotor retardation, without a special diagnosis, and for the last 4 months she has been hospitalized three times in Syria for failure to thrive and recurrent vomiting.

On admission, she was afebrile, with failure to thrive; her body parameters were as follows: weight: 7460 g (<3rd percentile), height: 75 cm (3rd–10th percentile), and head circumference PC: 44 cm (<3rd percentile). Clinical examination was unremarkable; no signs of child abuse or neglect were observed. A convergent strabismus was noticed, and neurological examination revealed psychomotor retardation. Laboratory tests were within normal limits, without anemia, hypoalbuminemia, or electrolyte disturbances. Thyroid function and immunological parameters were normal. There were no signs of infection; all cultures were negative. Because of the clinical picture and her history, a brain MRI and an upper GI series were ordered.

**Figure 1:**
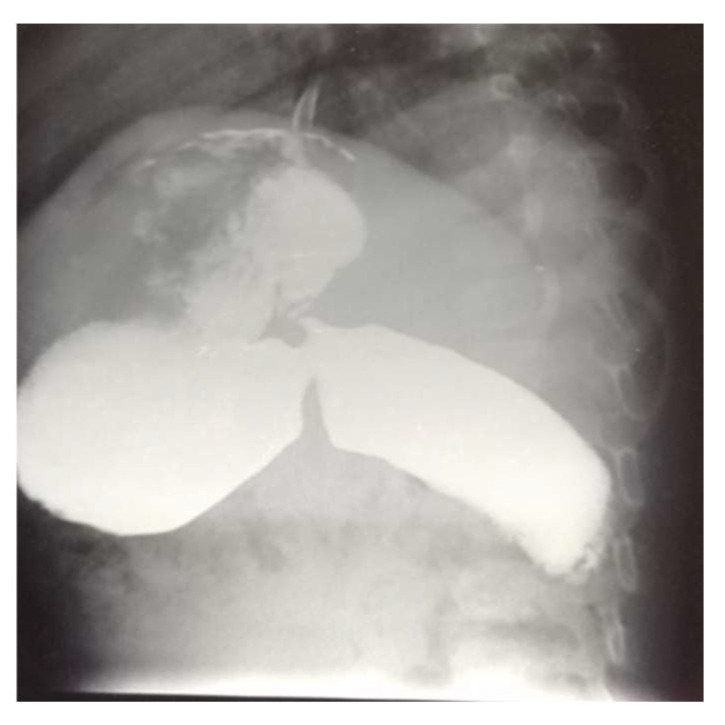
Long delay in the passage of the contrast medium in the second part of duodenum with a large degree of prostenotic dilatation.

**Figure 2:**
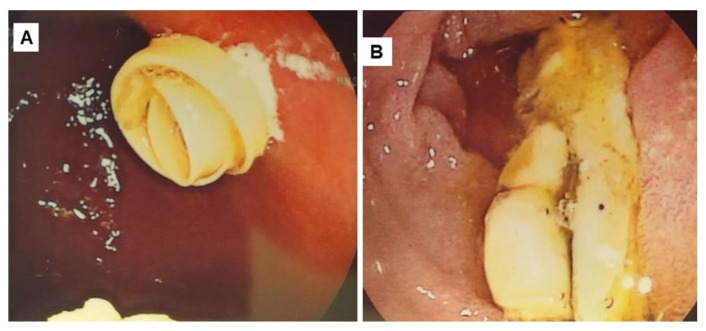
Endoscopy images; food debris in (**A**) stomach, (**B**) bulbus of duodenum.

**Figure 3:**
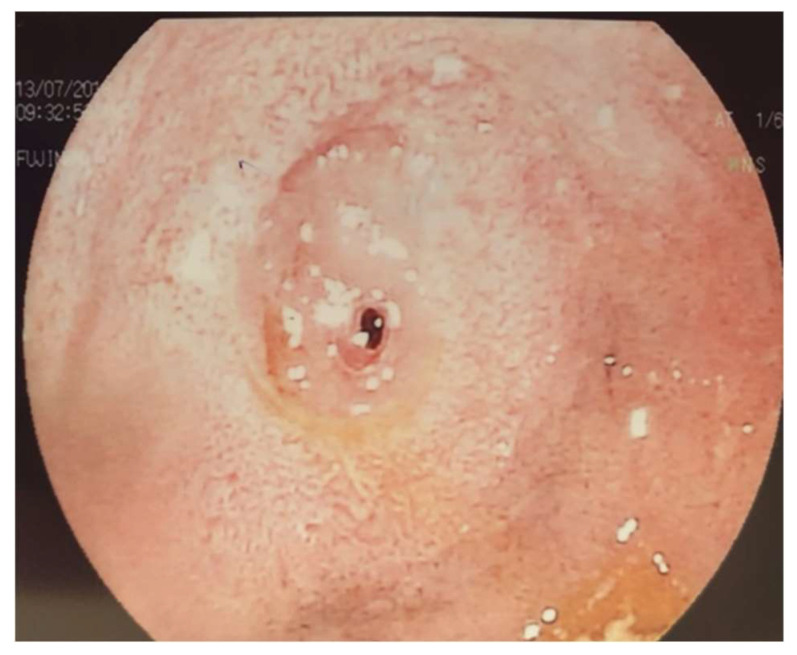
Endoscopy images; congenital duodenal diaphragm/web.

The brain MRI did not reveal pathological findings, while the upper GI series revealed a long delay in the passage of the contrast medium in the second part of the duodenum which was observed with a large degree of prostenotic dilatation (Figure 1). An upper GI endoscopy was performed, where gastritis and food debris were found in the stomach and duodenal bulb. In the second part of the duodenum (Figure 2), a congenital duodenal diaphragm was found (Figure 3).

Work-up for other coexistent malformations was negative. Surgical intervention was therefore decided, and a longitudinal duodenotomy with diaphragm resection followed by duodenoplasty and appendectomy were performed.

The full surgical report is as follows: A Kocher incision was made, followed by lay-er-by-layer dissection to enter the peritoneal cavity. The first and second portions of the duodenum were found to be dilated. A longitudinal duodenotomy was performed, and the diaphragm (septum) was identified, divided, and its two leaflets were sutured to the duodenal wall on both sides. The duodenal walls were closed using Gambee sutures. An appendectomy was performed without stump inversion. Meckel’s diverticulum was not identified. Layered closure was completed in anatomical order, placement of a Penrose drain, meticulous hemostasis, and the skin was closed with metallic clips; recovery was uneventful. The postoperative course was smooth, and the patient was discharged on postoperative day 7. On follow-up, she remained asymptomatic with adequate weight gain.

## 3. Discussion

Complete webs are typically present in the neonatal period with marked bilious vomiting and obstruction. However, partial (fenestrated) diaphragms may have a more insidious course, presenting later in infancy or early childhood, often with nonspecific symptoms such as intermittent vomiting and failure to thrive, without classic imaging signs of obstruction [9]. Sarkar et al. described a 2-year-old with persistent non-bilious vomiting and failure to thrive—but without overt obstructive signs—who was ultimately diagnosed by a barium study with a duodenal web.

A series reviewing delayed presentations emphasized that webs with central apertures may only manifest as failure to thrive and food intolerance even beyond infancy [1]. In our patient, similar features are evident—long-standing projectile vomiting since birth, failure to thrive, and delayed diagnosis—highlighting the value of high clinical suspicion and targeted imaging. In our patient, some other factors such as familial and socioeconomic may have played role in this diagnostic delay and also the child book with growth chat and growth parameters measurements are not available.

The upper GI series remains a cornerstone in detecting duodenal webs. It often reveals a dilated proximal duodenum and stomach with a narrowed distal lumen, commonly referred to as a “windsock” appearance when the web prolapses distally under peristalsis [10]. This classic radiologic pattern aligns well with our imaging findings: delayed contrast passage in the second part of the duodenum with marked prestenotic dilation. Endoscopy provides direct visualization and confirmation of the diaphragm, as demonstrated in our case.

Key differentials for chronic vomiting in infants/toddlers with failure to thrive include the following: pyloric stenosis, gastroesophageal reflux disease (GERD), malrotation with intermittent volvulus, intestinal motility disorders, and metabolic or neurologic etiologies. The differential diagnosis of intermittent vomiting in children appears in Table 1.

**Table 1:** Differential diagnosis of intermittent vomiting in children.

System/Category	Condition	Key Features
**Gastrointestinal**	**Cyclic vomiting syndrome (CVS)**	Sudden, stereotyped episodes of intense vomiting lasting hours to days; symptom-free periods in between; often family history of migraine
	**Gastroesophageal reflux disease (GERD)**	Recurrent post-feeding vomiting or regurgitation; more common in infants; may cause failure to thrive
	**Pyloric stenosis** (early intermittent → later persistent)	Non-bilious projectile vomiting in infants (2–8 weeks); starts intermittently but progresses; palpable mass
	**Intestinal malrotation with intermittent volvulus**	Bilious vomiting; abdominal pain/distension; intermittent due to intermittent torsion
	**Partial bowel obstruction (adhesions, strictures)**	Intermittent vomiting with abdominal pain/distension; history of surgery may be present
	**Constipation**	Intermittent vomiting due to fecal loading/obstruction; abdominal bloating, palpable mass
**Neurological**	**Migraine (abdominal or classic)**	Vomiting with episodic abdominal pain or headache, photophobia, pallor; family history of migraine
	**Increased intracranial pressure (e.g., brain tumor)**	Intermittent morning vomiting, headache, worse with position change; may have papilledema or neurologic signs
**Metabolic/Endocrine**	**Inborn errors of metabolism (e.g., urea cycle defects)**	Vomiting triggered by fasting or protein intake; metabolic acidosis, encephalopathy in severe cases
	**Adrenal insufficiency (Addison’s disease)**	Intermittent vomiting with fatigue, hypotension, weight loss, hyperpigmentation, electrolyte disturbances
**Renal**	**UTI or hydronephrosis (intermittent)**	Vomiting may accompany fever, flank pain, or be intermittent due to obstruction
**Psychiatric/Behavioral**	**Rumination syndrome**	Voluntary regurgitation and rechewing; typically post-meal in older children; absence of nausea
	**Psychogenic vomiting/Functional nausea**	Vomiting without organic cause; often related to stress or anxiety; more common in adolescents
**Other/Miscellaneous**	**Motion sickness**	Recurrent vomiting triggered by travel; improves with rest or antiemetics
	**Food allergy/intolerance (e.g., cow’s milk protein)**	Recurrent vomiting associated with certain foods; may include diarrhea, eczema, or failure to thrive
	**Medication-induced (e.g., antibiotics, iron, anticonvulsants)**	Vomiting appears during or after medication use; resolves with discontinuation

In this patient with chronic projectile vomiting and failure to thrive, gastrointestinal causes such as pyloric stenosis, intestinal malrotation, partial bowel obstruction, and GERD were considered. Neurological, metabolic, endocrine, and renal disorders, as well as psychogenic or behavioral causes and food intolerance or medication effects, were also evaluated. Imaging and endoscopy ultimately confirmed a congenital duodenal diaphragm as the underlying cause.

Historically, open surgical correction—via duodenotomy and excision of the web, duodenoplasty, or bypass procedures—was the standard of care [1]. In recent decades, endoscopic treatments have emerged as minimally invasive alternatives, especially in partial obstructions. These include endoscopic balloon dilation (EBD) and endoscopic membranotomy with various tools (e.g., insulated-tip knives, laser ablation, snare resection) [3,5,8].

Poddar et al. reported successful balloon dilations in three children (aged 2–9 years) with duodenal webs located distal to the ampulla, achieving relief in 2–4 sessions without complications [3].

A broader retrospective series (2019–2022) observed 10 patients (aged 15 days to 7 years) treated endoscopically (diaphragmotomy ± dilation). Outcomes were excellent: operative durations were brief, no major complications occurred, hospital stays averaged about one week, and follow-up (3–36 months) showed sustained resolution in most, with manageable restenosis [8].

In our patient, a transanastomotic nasojejunal feeding tube was not placed; however, current evidence supports the use of transanastomotic feeding tubes (TATs) in neonates with congenital duodenal obstruction as they facilitate earlier enteral feeding, reduce parenteral nutrition, and decrease central line requirements without increasing complications [11]. Other studies [12] report similar benefits, though most evidence is observational. Overall, TATs are strongly recommended to improve early post-operative nutrition, but the lack of randomized trials means they cannot yet be considered universally essential.

Our case, which involved surgical duodenotomy and web excision, also resulted in smooth recovery and catch-up growth, consistent with the favorable prognosis noted in both surgical and endoscopic approaches.

Surgical excision via duodenotomy remains the most definitive treatment, with high success and low recurrence but at the cost of greater invasiveness and longer recovery. Endoscopic balloon dilation offers good results in selected cases with thin or partially obstructing webs and is minimally invasive, though repeat procedures may be needed. ESD-like endoscopic resection provides a precise, less invasive option for older children and adults, but requires advanced expertise and carries a higher technical risk. Endoscopic electrocauterization with an IT-knife has shown promising outcomes while avoiding surgery, though evidence is limited and operator skill is crucial [1,2,3,4,5].

Post-correction, prognosis is generally excellent if there are no coexisting severe anomalies [1,8]. Both surgical and endoscopic modalities yield good functional recovery. In endoscopic series, recurrent stenosis occurred in a subset, requiring repeat endoscopic intervention; long-term monitoring is advised [8].

In our patient, the smooth postoperative course and weight gain are entirely in keeping with these observations.

Potential complications following treatment of congenital duodenal webs include post-operative adhesive obstruction and residual or recurrent stenosis, particularly after endoscopic dilation or partial membrane incision [1,3,6,7,13]. Endoscopic procedures may also cause bleeding, perforation, or thermal injury, especially with insulated-tip knife or scissor-forceps resections [2,5,8,14].

Recommended follow-up includes monitoring for vomiting, feeding difficulties, and weight gain, with targeted imaging or interval endoscopic reassessment if symptoms persist, to confirm adequate luminal patency and detect early complications [5,8,9,10].

## 4. Conclusions

Congenital duodenal diaphragm is a rare but significant cause of partial intestinal obstruction in infants and young children. It should be considered in the differential diagnosis of persistent or recurrent vomiting, particularly when accompanied by failure to thrive and unremarkable laboratory findings. As highlighted in this case, diagnosis can be delayed due to the non-specific and intermittent nature of symptoms, especially in cases of incomplete obstruction. Upper gastrointestinal contrast studies, complemented by endoscopic evaluation, are essential diagnostic tools for identifying this condition. While endoscopic treatment options are evolving, surgical correction through duodenotomy and septal resection remains the gold standard, offering rapid symptom relief and catch-up growth with low morbidity. This case underscores the importance of maintaining a high index of suspicion for congenital duodenal webs in young children with chronic vomiting and growth failure—even in the absence of classical signs of intestinal obstruction. Early diagnosis and timely intervention are critical to prevent long-term complications such as malnutrition, developmental delay, and gastrointestinal morbidity.

## Data Availability

The original contributions presented in this study are included in the article. Further inquiries can be directed to the corresponding author.

## Abbreviations

The following abbreviations are used in this manuscript:

CDD	Congenital duodenal diaphragm
GI	Gastrointestinal
FTT	Failure to thrive
ESD	Endoscopic submucosal dissection
IT	Insulated-tip
MRI	Magnetic resonance imaging
GERD	Gastroesophageal reflux disease
